# Genetic Manipulation of Non-Falciparum Human Malaria Parasites

**DOI:** 10.3389/fcimb.2021.680460

**Published:** 2021-08-30

**Authors:** Taís Baruel Vieira, Thafne Plastina Astro, Roberto Rudge de Moraes Barros

**Affiliations:** Department of Microbiology, Immunology and Parasitology, Escola Paulista de Medicina, Universidade Federal de São Paulo, São Paulo, Brazil

**Keywords:** transfection, reporter genes, drug selection, *Plasmodium knowlesi*, *Plasmodium vivax*, *Plasmodium cynomolgi*

## Abstract

The development of genetic manipulation of *Plasmodium falciparum* in the 1980s was key to study malaria biology. Genetically modified parasites have been used to study several aspects of the disease, such as red blood cell invasion, drug resistance mechanisms, gametocyte development and mosquito transmission. However, biological and genetic differences between *P. falciparum* and the other human malaria parasites make *P. falciparum* a poor model to study different species. The lack of robust systems of long-term *in vitro* culture of *P. vivax* and the other human malaria parasites lagged the genetic manipulation of these species. Here we review the efforts to generate genetically modified non-*falciparum* human malaria parasites, *in vivo* and *in vitro*. Using *in vivo* models – infection of non-human primates such as rhesus macaques and saimiri monkeys – researchers were able to generate transgenic lines of *P. knowlesi, P. cynomolgi*, and *P. vivax*. The development of long-term *in vitro* culture of *P. knowlesi* in the 2000’s, using rhesus and human red blood cells, created a platform to genetically manipulate non-*falciparum* malaria parasites. Recently, the use of CRISPR/Cas9 technology to genome edit *P. knowlesi* provides another tool to non-falciparum malaria research, extending the possibilities and allowing researchers to study different aspects of the biology of these parasites and understand the differences between these species and *P. falciparum*.

## Background

Bacterial transformation was discovered in 1955 (Hotchkiss, 1955) and since then scientists have been able to manipulate the genomes of several organisms using the same principle. The transformation mechanism itself was used first to manipulate bacterial genes (Stocker, 1963). In 1982, a group used electric pulses to transfer the herpes simplex *thymidine kinase* gene into mouse lyoma cells (Neumann et al., 1982). This was the first report of the use of electroporation to facilitate the incorporation of exogenous DNA by cells. The first protozoan parasite transfection was reported in 1987 when an exogenous plasmid was introduced in *Trypanosoma brucei* (Eid and Sollner-Webb, 1987). The first malaria parasite transfected was *Plasmodium gallinaceum* in 1993 (Goonewardene et al., 1993).

Since then, four of the six *Plasmodium* species that cause human malaria (*Plasmodium falciparum*, *Plasmodium vivax*, *Plasmodium knowlesi*, and *Plasmodium cynomolgi*) (Wu et al., 1995; Van Der Wel et al., 1997; Kocken et al., 1999; Barros et al., 2015) have been submitted to genetic transformation (van Dijk et al., 1995; Mota et al., 2001; Reece and Thompson, 2008). Different techniques were applied: integration of circular and linear DNA by homologous recombination (van Dijk et al., 1995; Crabb and Cowman, 1996; Mota et al., 2001; Kocken et al., 2002), Plasmodium Artificial Chromosomes (PACs) (Voorberg-van der Wel et al., 2013), and double-strand break based genome editing techniques, such as Zinc Finger Nucleases (ZFNs) (Straimer et al., 2012; Barros et al., 2015) and Clustered Regularly Interspaced Short Palindromic Repeats (CRISPR)/Cas9 (Ghorbal et al., 2014; Lee and Fidock, 2014; Wagner et al., 2014; Zhang et al., 2014).

Given its impact on economy and global health, robust methods for genetic analysis are needed to study malaria parasites. However, research is still focused on *P. falciparum*, species responsible for the majority of human deaths. Research of the other human malaria parasites (*P. vivax, P. knowlesi, P. cynomolgi, P. malariae* and *P. ovale*) remains neglected (Carlton et al., 2011), including the development of transgenic parasites. In this paper we review all the published papers on genetic transformation of non-falciparum human malaria parasites, a field that should grow exponentially with the recent development of *in vitro* culture of *P. knowlesi* and *P. cynomolgi* (Kocken et al., 2002; Moon et al., 2013; Chua et al., 2019) combined with the use of genome editing techniques.

## Plasmodium knowlesi

Historically, *P. knowlesi* has been extensively used as a model for malaria research because their natural and experimental hosts (*Macaca fascicularis* and *Macaca mulatta*, respectively) were easily available for research. However, the development of *in vitro* culture of *P. falciparum* and the restrictions to the use of non-human primates shifted the focus from *P. knowlesi* research in the 1970s (Butcher and Mitchell, 2016; Pasini et al., 2016). Transfection of *P. knowlesi* was first described in 1997. The authors developed a parasite line resistant to the antifolate pyrimethamine, using an episomal construct containing the *Toxoplasma gondii dihydrofolate reductase-thymidylate synthase* gene (*tgdhfr-ts*). The episomal plasmids also harbored expression control sequences originally from *P. berghei* and *P. falciparum* (Van Der Wel et al., 1997). Transgenic parasites were obtained *in vivo*, after the inoculation of electroporated parasitized red-blood cells in rhesus macaques. The results demonstrated that *P. knowlesi* parasites recognize expression control sequences from evolutionary distant malaria species, such as the rodent malaria *P. berghei* and the laverania malaria *P. falciparum.*

*Plasmodium knowlesi* was successfully adapted to long term *in vitro* culture using rhesus red-blood cells in 2001 (Kocken et al., 2002). Culture adapted parasites could be used later for rhesus infections, allowing studies *in vitro* and *in vivo*. Furthermore, parasites were transfected transiently or stably *in vitro*, using circular episomes or genomic integration of linearized DNA by single or double crossover (Kocken et al., 2002; Ozwara et al., 2003). The adaptation of *Plasmodium knowlesi* to culture using human red-blood cells in 2013 was a key advancement for non-falciparum malaria research (Moon et al., 2013). The need for expensive animal infections or rhesus blood was eliminated, facilitating parasite maintenance and allowing the use of selective agents potentially toxic to animal models. The authors also presented results of parasite transfections, showing fast and highly effective development of transgenic parasites, using GFP as reporter gene and *human dhfr* (*hdhfr*) as selective marker (Moon et al., 2013).

One of the major challenges for Plasmodium transfection have been the use of reliable selection markers. A limited number of drugs can be used as selective agents, especially *in vivo*, where pyrimethamine is the only drug available to select transgenic parasites in non-human primates. The first paper to address this issue was published in 2004 (Wel et al., 2004), when the authors evaluated the sensitivity of *P. knowlesi* to drugs previously used to select transgenic *P. falciparum*: Blasticidin S, G418 and WR99210. The genes that confer resistance to these drugs are *blasticidin S deaminase* (*bsd*), *neomycin phosphotransferase II* (*neo*) and human *dihydrofolate reductase* (*hdhfr*), respectively. The authors concluded that these genes can be used as positive selective markers for transfected parasites. However, drug concentrations of Blasticidin necessary to inhibit growth of *P. knowlesi in vitro* were 20-fold higher than the ones used to select transgenic *P. falciparum*, limiting the use of this drug (Wel et al., 2004). Several years later, in 2017, a study presented the antiplasmodial activity against *P. knowlesi* and *P. falciparum* of three compounds used for transgenic parasite selection *in vitro*: Blasticidin, the antifolate WR99210 and DSM-1. Results confirmed that *P. knowlesi* is naturally less sensitive to BSD than *P. falciparum*, and also showed that DSM-1 is required in a concentration at least 3-fold higher to inhibit *P. knowlesi* growth when compared to *P. falciparum* (van Schalkwyk et al., 2017).

Different methods can be used to genetic transform *Plasmodium* parasites, such as electroporation of parasitized red-blood cells or transformation by spontaneous plasmid uptake by *P. falciparum* after invasion of electroporated uninfected red-blood cells (Deitsch et al., 2001). This technique was applied to *P. knowlesi* in 2017, providing a convenient and unexpensive method to generate transgenic parasites. The study also showed that late-stage schizonts are mandatory for high electroporation efficiency of parasitized red-blood cells (Moraes Barros et al., 2017).

Genome editing of *P. knowlesi* using the CRISPR/Cas9 technology was achieved in 2019 (Mohring et al., 2019). The authors tested two different strategies to deliver the donor sequence for homologous recombination. Parasites were transfected with one plasmid harboring the genes encoding Cas9, the sgRNA, and the selective markers. The donor sequences were provided in a second plasmid or a linear DNA construct, obtained by PCR. Both approaches successfully generated transgenic parasites with high efficiency, with stable lines observed in 12 days. The use of linear PCR constructs as donor sequences is a fast and reliable method for gene tagging and knockout. The group also defined optimal parameters and limits to the CRISPR/Cas9 technique in *P. knowlesi.* Observing integration rate and parasite culture recovery period after drug pressure, results suggest that the optimal size for homology regions is between 200 bp and 800 bp. As for the distance between the homology regions and the double strand break site, integrations efficiency varies depending on the size of the homologous region - larger homologous regions allow more distant DSB sites. So larger constructs require larger homologous sequences for successful DSB repair. The authors concluded that the biggest bottleneck in the CRISPR/Cas9 system is the parasite’s homologous recombination mediated DNA repair system - as the majority of Cas9 induced DSBs are not successfully repaired (Mohring et al., 2019).

Recently, *P. knowlesi* parasites were genetic modified *in vitro* using circular plasmids containing *P. vivax* centromeric repeats. The presence of centromeric sequences of *P. vivax* provided higher stability to the episomes, allowing faster selection of transgenic parasites and the maintenance of the plasmid throughout the complete life cycle, *in vitro* and *in vivo*. The paper also presented for the first time that *P. vivax* regulatory sequences not recognized by *P. falciparum* are recognized by *P. knowlesi*, providing an *in vitro* model for *P. vivax* genetic studies. A transgenic line developed in this work expresses the reporter gene nanoluc and can be used for drug activity studies against blood stage and liver stage parasites and transmission blocking studies (Barros et al., 2021).

## Plasmodium cynomolgi

Transfection of *P. cynomolgi* was reported for the first time in 1999, using parameters and DNA constructs similar to the ones used to transform *P. knowlesi* in 1997 (Kocken et al., 1999). After this first report, no paper was published presenting results of *P. cynomolgi* transformation until 2013, when this species was transformed using Plasmodium Artificial Chromosomes (PACs) (Voorberg-van der Wel et al., 2013).

In 2010, Iwanaga et al. (2010) identified and characterized the *Plasmodium* centromere and constructed a circular *P. berghei* artificial centromere (PAC) containing the new-found centromere sequences and also a linear *P. berghei* artificial centromere (L-PAC), containing the centromeric and telomeric repeats. Unlike transfections with circular episomal DNA, transfections with PAC are capable of stable segregation without drug selective pressure, remaining stable throughout the parasite’s life cycle (Iwanaga et al., 2010; Barale and Ménard, 2010). PACs containing *P. cynomolgi* centromeric repeats were designed in 2013 to genetic modify *P. cynomolgi* parasites *in vivo*, allowing the development of a transgenic line expressing reporter gene (GFP) throughout the parasite’s life cycle, including hypnozoites. This transgenic line can be used to analyze reactivation conditions and for high throughput drug screening against liver stage parasites (Voorberg-van der Wel et al., 2013). PACs containing the GFP gene under control of the *heat shock 70* gene (*pchsp70)* promoter sequence were used to transform *P. cynomolgi* in 2020, allowing real time visualization of hypnozoite reactivation (Voorberg-van der Wel et al., 2020). In addition to that, a second reporter gene (mCherry) was added to the PAC, under transcriptional control of the schizont-specific promoter sequence of the *liver-specific protein 2* (*lisp2*) gene, highlighting the parasites that had initiated schizogony (Voorberg-van der Wel et al., 2020). To this date, all *P. cynomolgi* transfections were performed *in vivo*, using macaque infections and pyrimethamine as selection agent (Kocken et al., 1999; Voorberg-van der Wel et al., 2013; Voorberg-van der Wel et al., 2020).

## Plasmodium vivax

The first transfection experiments of *P. vivax* were reported in 2005, transient transfection of parasites with episomal plasmids containing the luciferase reporter gene and *P. falciparum* regulatory sequences (Pfahler et al., 2006). No advancements were made in *P. vivax* transfection until 2015, when the genome of this species was manipulated for the first time, *in vivo* (Barros et al., 2015). Using the genome editing technique of Zinc-Finger Nucleases, that creates DNA double strand-breaks at specific sites, the authors were capable to insert point mutations in the *P. vivax dhfr*-ts gene, transforming parasites sensitive to pyrimethamine in resistant to this drug. This was the first report of use of genome editing techniques in non-falciparum *Plasmodium*, demonstrating the possibility of site-specific genome editing (Barros et al., 2015). The experiments were performed *in vivo*, using *Saimiri boliviensis* monkeys. The resistant/transfected parasites were selected for three weeks in animals treated with pyrimethamine in a method similar to the one used to select transgenic *P. knowlesi* (Van Der Wel et al., 1997) and *P. cynomolgi in vivo*, using rhesus macaques (Kocken et al., 1999; Voorberg-van der Wel et al., 2013; Voorberg-van der Wel et al., 2020).

## Conclusions

*Plasmodium vivax*, *P. malariae*, *P. ovale*, *P. knowlesi* and *P. cynomolgi* are phylogenetically grouped together in the Primate malaria clade, evolutionary distant from *P. falciparum*, grouped with *P. reichenowi* and *P. gabonii* in the Laverania malaria clade (Loy et al., 2017). The large evolutionary distance between *P. falciparum* and the other human malaria parasites helps to explain the biological differences in the species, and makes mandatory specific genetic studies for non-falciparum human malaria species. Genetic manipulation is key to elucidate different aspects of these parasites’ biology, and these studies have been neglected for several years.

Transfection of non-falciparum human Plasmodium was reported for the first time in 1997, and since then only 12 papers were published describing new results using this technology. More than 20 years after the first transfection of *P. knowlesi* we have barely scratched the surface of what can be performed using these techniques. Little is known about specific processes of these parasites, such as red blood cell invasion, liver stage development and gametocyte biology. The first paper to use genetic modification to study specific features of non-falciparum human malaria parasites was published in 2019 when the authors used CRISPR/Cas9 to edit the *duffy-binding protein* (*pkdbp-1*) gene and were able to evaluate its role in red-blood cell invasion (Mohring et al., 2019). The **Table 1** summarizes the techniques used, selectable markers and genes targeted.

**Table 1:** Summary of techniques used in non-falciparum Plasmodium transfection studies.

Species	Techniques used	Selectable markers	Genes targeted
*Plasmodium knowlesi*	Episomal DNA (*in vivo*) (Van Der Wel et al., 1997)	*tgdhfr-ts* (Van Der Wel et al., 1997)	*pkp230p* (Mohring et al., 2019)	
	Integration by double crossover (*in vitro*) (Kocken et al., 2002)	*hdhfr* (Wel et al., 2004)	*pkama1* (Mohring et al., 2019)	
	Transformation by spontaneous DNA uptake (*in vitro*) (Moraes Barros et al., 2017)	*bsd* (Wel et al., 2004)	*Pkmyoa* (Mohring et al., 2019)	
	CRISPR/Cas9 (*in vitro*) (Mohring et al., 2019)	*neo* (Wel et al., 2004)	*pkk13 (*Mohring et al., 2019)	
	Centromeric plasmid (*in vitro*) (Barros et al., 2021)		*pkcrt* (Mohring et al., 2019)	
			*pkdbp* (Mohring et al., 2019)	
*Plasmodium cynomolgi*	Episomal DNA (*in vivo*) (Kocken et al., 1999)	*tgdhfr-ts* (Kocken et al., 1999)	None	
	Artificial Chromosome (*in vivo*) (Voorberg-van der Wel et al., 2013)	*hdhfr* (Voorberg-van der Wel et al., 2013)		
*Plasmodium vivax*	Transient episomal DNA (*in vivo*) (Pfahler et al., 2006)	*pvdhfr* with point mutations (Barros et al., 2015)	*pvdhfr* (Barros et al., 2015)	
	Zinc Finger Nucleases (*in vivo*) (Barros et al., 2015)			

*Plasmodium knowlesi* seems to be a formidable research model for members of the Primate Malarias clade. This species can be maintained *in vitro*, with a 24-hour assexual life cycle that provides rapid growth and fast generation of transgenic parasites. *In vitro* cultivated parasites can be used for blood stage, liver stage and transmission studies without the need of animal models (Grüring et al., 2014; Armistead et al., 2018). Furthermore, this parasite recognizes regulatory sequences such as transcriptional promoters and centromeric repeats of *P. vivax*, *P. falciparum* and *P. berghei*, allowing comparative genetic studies of different malaria species (Ozwara et al., 2003; Pasini et al., 2016; Barros et al., 2021).

The biggest bottleneck to non-falciparum malaria studies was the need for non-human primate infections and blood, but the development of long term *in vitro* culture of *P. knowlesi* using human red-blood cells changed the landscape (Moon et al., 2013). The recent development of *P. cynomolgi in vitro* culture is an exciting achievement (Chua et al., 2019), allowing specific studies of liver stage parasites, specially hipnozoites, that are not observed in *P. falciparum* and *P. knowlesi*, the other species that can be cultivated *in vitro*. Laboratories across the globe will also benefit from the success of new technologies that increase the efficiency of genetic transformation, such as artificial chromosomes and genome editing techniques. A second challenge that must be targeted for the expansion of Plasmodium transfection studies is the development of new selection markers, limited to antifolates. CRISPR derived techniques facilitate genetic knockout and knockdown and should be used to generate these transgenic lines. However, to improve the methods of genetic modification and create platforms of consecutive knockdowns of functional genes, new selection markers are necessary.

In the next few years, we hope that different genes and pathways will be the targets of genetic modification in non-falciparum malaria parasites, allowing a better comprehension of specific features of the biology of these parasites, leading to the development of better mechanisms of control and treatment of non-falciparum human malaria.

## Author Contributions

RRMB has contributed designing and writing the manuscript. TBV and TPA have contributed writing this mini-review. All authors contributed and approved the submitted version.

## Funding

This work has been supported by FAPESP grants JP 2018/06219-8 (RRMB), 2019/07223-1 (TPA), 2020/02303-4 (TBV) and CNPq 434011/2018-5 (RRMB).

## Conflict of Interest

The authors declare that the research was conducted in the absence of any commercial or financial relationships that could be construed as a potential conflict of interest.

## Publisher’s Note

All claims expressed in this article are solely those of the authors and do not necessarily represent those of their affiliated organizations, or those of the publisher, the editors and the reviewers. Any product that may be evaluated in this article, or claim that may be made by its manufacturer, is not guaranteed or endorsed by the publisher.
